# Supplementary material for: Venomix: a simple bioinformatic pipeline for identifying and characterizing toxin gene candidates from transcriptomic data
Source: PeerJ. 2018 Jul 31;6:e5361. doi: 10.7717/peerj.5361 (PMC6074769; doi:10.7717/peerj.5361)
Supplement: Supplemental Information 3 [file peerj-06-5361-s003.gz › FinalOutput_E-6/Kunitz-type_serine_protease_inhibitor_PIVL_13/finaltree.pdf]

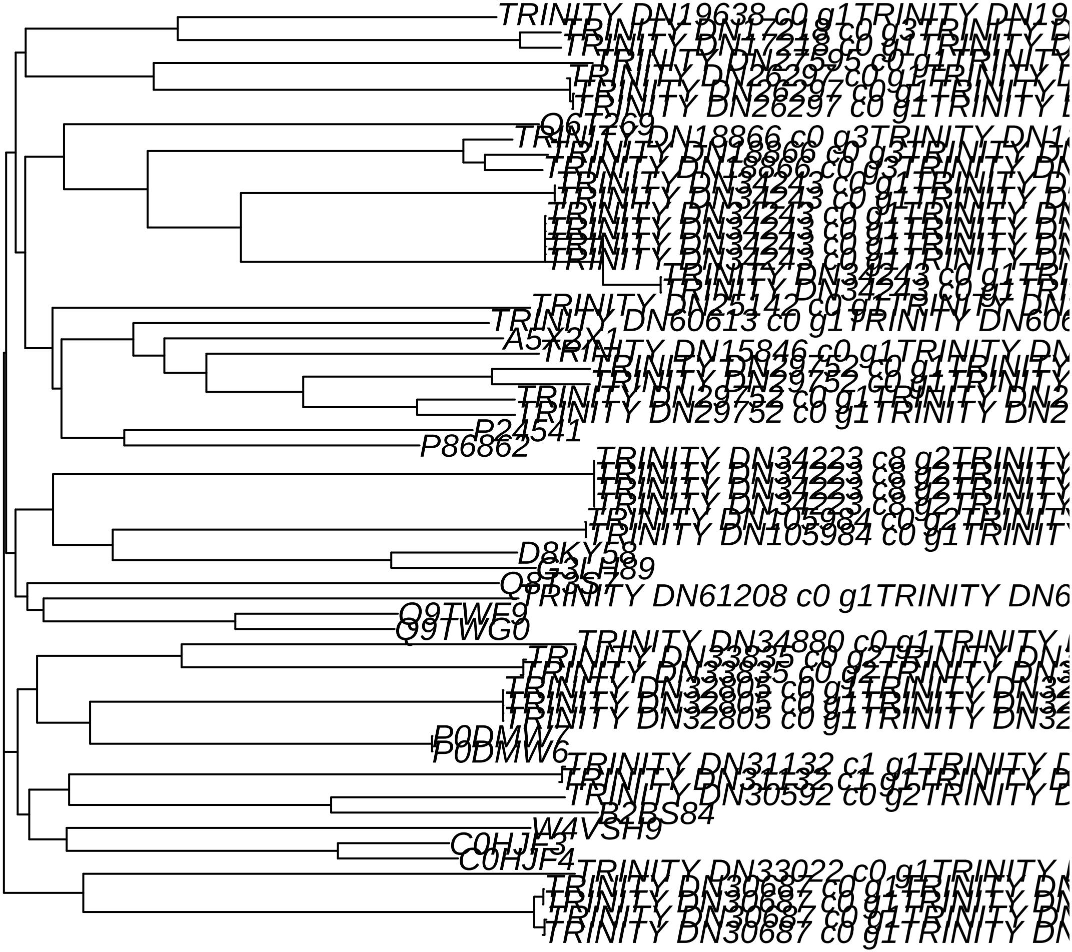
TRINITY DN19638 c0 g1TRINITY DN19
TRINITY DN17218 c0 g3TRINITY D
TRINITY DN17218 c0 g1TRINITY D
TRINITY DN27595 c0 g1TRINITY
TRINITY DN26297 c0 g1TRINITY D
Q6T269
TRINITY DN18866 c0 g3TRINITY DN1
TRINITY DN34243 c0 g1TRINITY D
TRINITY DN25142 c0 g1TRINITY DN
TRINITY DN60613 c0 g1TRINITY DN60
A5X2X1
TRINITY DN15846 c0 g1TRINITY DN
TRINITY DN29752 c0 g1TRINITY DN2
P24541
P86862
TRINITY DN34223 c8 g2TRINITY
TRINITY DN105984 c0 g2TRINITY
TRINITY DN105984 c0 g1TRINITY
D8KY58
G3LH89
Q8T3S7
TRINITY DN61208 c0 g1TRINITY DN6
Q9TWF9
Q9TWG0
TRINITY DN34880 c0 g1TRINITY
TRINITY DN33835 c0 g2TRINITY DN3
TRINITY DN32805 c0 g1TRINITY DN32
P0DMW7
P0DMW6
TRINITY DN31132 c1 g1TRINITY D
TRINITY DN30592 c0 g2TRINITY D
B2BS84
W4VSH9
C0HJF3
C0HJF4
TRINITY DN33022 c0 g1TRINITY
TRINITY DN30687 c0 g1TRINITY DN
